# Genetic Regulation of the Thymic Stromal Lymphopoietin (TSLP)/TSLP Receptor (TSLPR) Gene Expression and Influence of Epistatic Interactions Between IL-33 and the TSLP/TSLPR Axis on Risk of Coronary Artery Disease

**DOI:** 10.3389/fimmu.2018.01775

**Published:** 2018-08-03

**Authors:** Shao-Fang Nie, Ling-Feng Zha, Qian Fan, Yu-Hua Liao, Hong-Song Zhang, Qian-Wen Chen, Fan Wang, Ting-Ting Tang, Ni Xia, Cheng-Qi Xu, Jiao-Yue Zhang, Yu-Zhi Lu, Zhi-Peng Zeng, Jiao Jiao, Yuan-Yuan Li, Tian Xie, Wen-Juan Zhang, Dan Wang, Chu-Chu Wang, Jing-Jing Fa, Hong-Bo Xiong, Jian Ye, Qing Yang, Peng-Yun Wang, Sheng-Hua Tian, Qiu-Lun Lv, Qing-Xian Li, Jin Qian, Bin Li, Gang Wu, Yan-Xia Wu, Yan Yang, Xiang-Ping Yang, Yu Hu, Qing K. Wang, Xiang Cheng, Xin Tu

**Affiliations:** ^1^Department of Cardiology, Union Hospital, Tongji Medical College, Huazhong University of Science and Technology, Wuhan, China; ^2^Key Laboratory for Biological Targeted Therapy of Education Ministry and Hubei Province, Union Hospital, Tongji Medical College, Huazhong University of Science and Technology, Wuhan, China; ^3^Innovation Institute, Huazhong University of Science and Technology, Wuhan, China; ^4^Department of Cardiology, Zhongnan Hospital of Wuhan University, Wuhan, China; ^5^Department of Molecular Cardiology, Cleveland Clinic Lerner Research Institute, Cleveland, OH, United States; ^6^Key Laboratory of Molecular Biophysics of Ministry of Education, College of Life Science and Technology, Center for Human Genome Research, Cardio-X Institute, Huazhong University of Science and Technology, Wuhan, China; ^7^Department of Endocrinology, Union Hospital, Tongji Medical College, Huazhong University of Science and Technology, Wuhan, China; ^8^Department of Geriatrics, the Central Hospital of Wuhan, Tongji Medica College, Huazhong University of Science and Technology, Wuhan, China; ^9^Section of Molecule Medicine, Department of Medicine, University of Oklahoma Health Sciences Center, Oklahoma City, OK, United States; ^10^Jining Medical College Affiliated Hospital, Jining, China; ^11^Suizhou Central Hospital, Suizhou, China; ^12^Xiangyang Central Hospital, Xiangyang, China; ^13^Renmin Hospital of Wuhan University, Wuhan, China; ^14^Wuhan No. 1 Hospital, Wuhan, China; ^15^School of Basic Medicine, Tongji Medical College, Huazhong University of Science and Technology, Wuhan, China; ^16^Institute of Hematology, Union Hospital, Tongji Medical College, Huazhong University of Science and Technology, Wuhan, China

**Keywords:** thymic stromal lymphopoietin/TSLP receptor, IL-33, epistatic, coronary artery disease, genetic regulation

## Abstract

The thymic stromal lymphopoietin (TSLP)/TSLP receptor (TSLPR) axis is involved in multiple inflammatory immune diseases, including coronary artery disease (CAD). To explore the causal relationship between this axis and CAD, we performed a three-stage case-control association analysis with 3,628 CAD cases and 3,776 controls using common variants in the genes *TSLP, interleukin 7 receptor* (*IL7R*), and *TSLPR*. Three common variants in the TSLP/TSLPR axis were significantly associated with CAD in a Chinese Han population [rs3806933^T^ in *TSLP, P*_adj_ = 4.35 × 10^−5^, odds ratio (OR) = 1.18; rs6897932^T^ in *IL7R, P*_adj_ = 1.13 × 10^−7^, OR = 1.31; g.19646A>G^A^ in *TSLPR, P*_adj_ = 2.04 × 10^−6^, OR = 1.20]. Reporter gene analysis demonstrated that rs3806933 and rs6897932 could influence *TSLP* and *IL7R* expression, respectively. Furthermore, the “T” allele of rs3806933 might increase plasma TSLP levels (*R*^2^ = 0.175, *P* < 0.01). In a stepwise procedure, the risk for CAD increased by nearly fivefold compared with the maximum effect of any single variant (*P*_adj_ = 6.99 × 10^−4^, OR = 4.85). In addition, the epistatic interaction between *TSLP* and *IL33* produced a nearly threefold increase in the risk of CAD in the combined model of rs3806933^TT^-rs7025417^TT^ (*P*_adj_ = 3.67 × 10^−4^, OR = 2.98). Our study illustrates that the TSLP/TSLPR axis might be involved in the pathogenesis of CAD through upregulation of mRNA or protein expression of the referenced genes and might have additive effects on the CAD risk when combined with IL-33 signaling.

## Introduction

Genetic factors have been demonstrated to be equally important as environmental factors in the pathogenesis of coronary artery disease (CAD). Based on family and twin studies, researchers have estimated that the heritability of CAD is between 40 and 60% (1). Much progress has been made in this area. Currently, about 95 common risk variants of CAD have been detected by genome-wide association studies (GWASs), and a total of 202 independent signals in 109 risk loci have been discovered using genome-wide complex trait analysis software, which together explain more than 28% of the estimated heritability of CAD (2–5). The remaining unexplained heritability may be due to many reasons, including that the diverse characteristics of the commercial chips used in GWASs, the problem of genome-wide statistical significance level, epistatic interactions (epistasis), epigenetic influences, etc. (6, 7). Therefore, identification of causal genes or SNPs for complex diseases such as CAD by functional studies has become a hotspot for research worldwide.

The thymic stromal lymphopoietin (TSLP)/TSLP receptor (TSLPR) axis plays an important role in the regulation of a broad spectrum of inflammatory immune response-related diseases, including asthma and CAD. The key proteins of this axis are as follows: TSLP, a four-helix bundle cytokine targeting a variety of inflammatory immune cells, such as dendritic cells (DCs), B cells, mast cells, regulatory T cells, and CD4^+^ and CD8^+^ T cells (8–12); and the TSLPR complex, which consists of the IL-7 receptor alpha (IL-7Rα) and the unique TSLPR subunit (TSLPR; also known as CRLF2) (13–15). Under the stimulation of oxidized low-density lipoprotein (ox-LDL), human umbilical vein endothelial cells and vascular smooth muscle cells release large amounts of TSLP, which might activate DCs and then accelerate the development of atherosclerosis (16). TSLPR may also be expressed on human platelets and plays a role in the activation of platelets in acute coronary syndrome (17). Most recently, Li et al. identified that variants in *TSLP* might be involved in the development of asthma *via* regulation of the expression of *TSLP* (18). These results indicated that it is likely that causal SNPs or genes in the TSLP/TSLPR axis for CAD also exist. However, previous reports that addressed the role of the TSLP/TSLPR axis in CAD and atherosclerosis have yielded inconsistent results. In *in vitro* experiments, Lin et al. and Zhao et al. reported that TSLP can induce Th17 cell differentiation (16, 19). However, until now, the effects of Th17 cells and IL-17 on atherosclerosis remain unclear (20). In *in vivo* experiments with ApoE^−/−^ mice, Yu et al. found that the aortic root of mice treated with TSLP and TSLP-expressing DCs developed fewer atherosclerotic plaques than did control mice, suggesting a protective role for TSLP in CAD progression (21). However, more recently, Wu et al. reported that fewer arterial lesions developed in TSLPR-chain deficient ApoE-double knockout mice (ApoE-TSLPR DKO) than that did in ApoE knockout mice, indicating that the TSLP/TSLPR axis might promote the development of CAD (22).

Therefore, we performed the following studies to uncover the potential causal genes for the influence of the TSLP/TSLPR axis in the pathogenesis of CAD: (1) selecting all the tag SNPs covering the key genes of the TSLP/TSLPR axis; (2) performing a three-stage case-control genetic association study for CAD based on Chinese Han population with a large sample size; the selected tag SNPs were tested in stage 1 discovery study and then the positive associations from stage 1 discovery study were further tested in stage 2 validation study and stage 3 replication study; (3) testing correlations between the significant variants for CAD and the expression levels of their genes, by a reporter gene analysis or a circulation level study; and (4) constructing the interaction model and detecting the effect size of the causal variants in the contribution to the development of CAD.

Though common variants themselves may have very low effects on the pathogenesis of diseases, the epistasis (epistatic gene interaction) between common variants in the genes regulated by each other might have quite different functions in populations with different genetic backgrounds, thereby leading to a quite different effect size or direction on the risk of the diseases. In 2013, we reported that variants in the IL-33 signaling pathway might influence the development of CAD by regulating the expression levels of key genes of the pathway (23). The Framingham Heart Study identified that five missense variants in the *IL1RL1* gene, which encodes the receptor of IL-33, might influence the circulation level of soluble ST2 (sST2). The exact mechanism might be that the missense variants in *IL1RL1* regulate the expression of *IL33* and the interaction between IL-33 and its receptor ST2L, and then significantly increase the circulating level of sST2 (24). Other studies found that the TSLP and IL-33 signaling pathways might interact with each other in Th2 cell-mediated inflammatory responses (25–27).

These results indicated that variations in genes in the IL-33 and TSLP signaling pathways might contribute to epistatic interactions and then affect the develop of conditions such as CAD. In the present study, we investigated the potential epistatic interaction between *IL33* and *TSLP* in the pathogenesis of CAD.

## Materials and Methods

### Study Populations

The total sample size was 7,404, with 3,628 CAD cases and 3,776 controls (GeneID database) (23, 28–30). The discovery cohort in stage 1 was composed of 1,345 cases and 1,156 controls (total of 2,501 subjects). The validation cohort in stage 2 was composed of 1,347 cases and 1,156 controls (total of 2,503 subjects). The replication cohort in stage 3 was composed of 936 cases and 1,464 controls (total of 2,400 subjects). The inclusion and exclusion criteria for the CAD patients and controls were described previously (23, 28–30).

This study followed the guidelines set forth by the Declaration of Helsinki and passed the review of the Ethics Committee of Tongji Medical College, and Huazhong University of Science and Technology. All study participants have signed a written informed consent form.

### Genetic Analysis

**Figure 1:**
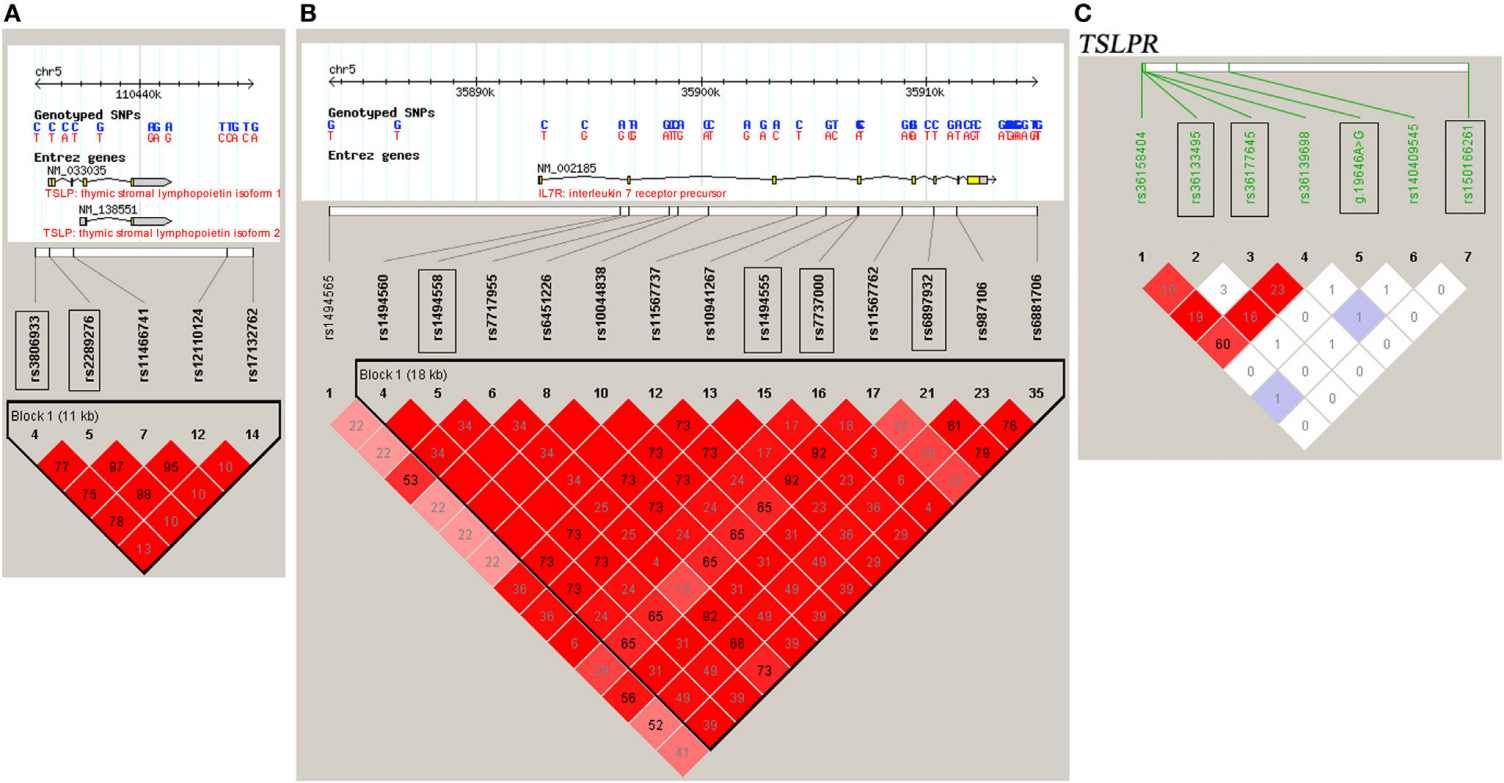
Linkage disequilibrium (LD) blocks of thymic stromal lymphopoietin (*TSLP*) and interleukin 7 receptor (*IL7R*) and TSLP receptor (*TSLPR*). The LD blocks of **(A)** *TSLP* and **(B)** *IL7R* were constructed based on the HapMap CHB and JPT datasets, and the LD blocks of **(C)** *TSLPR* were constructed based on the data obtained by direct DNA sequencing of all the exons, the 500 bp of the 5′ flanking region and the intron–exon junctions of the gene *TSLPR* in 50 coronary artery disease cases and 50 healthy controls. The selected tag SNPs were marked with rectangular boxes. Each diamond represents the LD degree between the SNPs. The color indicates the *D*′ (a redder color represents a higher *D*′) and the numbers within the diamonds are the *r^2^* values.

DNA samples were obtained using a Wizard Genomic DNA Purification Kit (Promega Corporation, Madison, WI, USA). A High-Resolution Melt system with Rotor-gene 6000 software (Corbett Life Science) was used for genotyping, which was validated by direct DNA sequencing analysis (23, 28–30). Two positive controls for each genotype were included in each run. For each SNP, a total of 48 cases and controls were randomly selected for verification of genotyping results using direct DNA sequencing analysis. Two principals were applied to select the tag SNPs (23, 28–30): (1) the threshold of *r*^2^ or *D*′ was more than 0.8 between the SNPs [Haploview (v.4.2), based on HapMap CHB and JPT datasets for *TSLP* and interleukin 7 receptor (*IL7R*) (v.3, release 2) or on direct sequencing data for *TSLPR*]; (2) the threshold of the minor allele frequency was greater than 0.05. Tag SNPs, such as rs3806933 and rs2289276 in *TSLP* and rs1494558, rs1494555, rs7737000, and rs6897932 in *IL7R* were selected (Figures 1A,B). For *TSLPR*, we detected the common variants by direct DNA sequencing of all the exons, the 500 bp of the 5′ flanking region and the intron–exon junctions of the gene in 50 CAD cases and 50 healthy controls. Among the identified 18 variants (Table S1 in Supplementary Material), 4 variants (rs150166261, rs36133495, rs36177645, and g.19646A>G) in *TSLPR* were selected for the association analysis (Figure 1C).

### Reporter Gene Assay

We performed the reporter gene assay to investigate whether the variants could regulate their gene expression. For the promoter variant rs3806933 in *TSLP*, two plasmids of TSLP-C and TSLP-T were generated with the pGL3-basic vector (23). In addition, 500 ng of each plasmid, along with 50 ng of a pRL-TK vector (Promega), were transfected into the human embryonic kidney 293 (American Type Culture Collection) cell line using Lipofectamine™ 2000 reagent (Invitrogen). Then, we measured the firefly luciferase activity using a Dual Luciferase Reporter Assay Kit (Promega) at 48 h after transfection. Three independent experiments were carried out with the empty pGL3-basic vector, and each was carried out in triplicate. For rs6897932 in *IL7R* and g.19646A>G in *TSLPR*, the reporter gene assay experiments were carried out with the pGL3-control vector (Promega) in the human HELA cell line, and three independent experiments were performed.

### Plasma Level of TSLP in CAD Patients

A total of 336 CAD patients were selected from the replication cohort for analysis of the association between rs3806933 genotypes and the plasma level of TSLP. The plasma samples were frozen at –80°C and tested in less than 2 months for TSLP concentration with an enzyme-linked immunosorbent assay kit (eBioscience Inc.).

### Genetic Risk Score (GRS) Analysis

Genetic risk scores were analyzed using a Cox regression model (SPSS version 17.0, SPSS, Inc., Chicago, IL, USA), adjusting for the conventional risk factors of CAD to obtain survival forecasts and calculate the C indices. GRS1 comprised SNP rs3806933 of *TSLP*; GRS2 comprised SNP rs7025417 of *IL33*; and GRS3 comprised the both SNPs rs3806933 of *TSLP* and rs7025417 of *IL33*.

### Statistical Analysis

All the control cohorts were examined using the Hardy–Weinberg equilibrium (HWE) test (PLINK version 1.06). The allelic and genotypic association analyses were performed using chi-square tests with Pearson’s 2 × 2 and 2 × 3 contingency tables (SPSS version 17.0). Under the genotypic model, the interaction analysis was carried out by a logistic association analysis with the conventional risk factors for CAD as covariates (23). Statistical power analyses were performed with a free power and sample size calculation program (PS v.3.0.12) for single gene association analysis and a free software QANTO (QANTO V.1.2.4) for interaction analysis (23). The difference in the luciferase activity among the reference plasmids was analyzed using the ANOVA test. The correlations among the rs3806933 genotypes and TSLP plasma concentrations were studied by linear regression analysis and the ANOVA test. The terminology of *P* < 0.01 in the text, figures, and figure legends indicates statistical significance.

## Results

### Population Characteristics

The CAD patients were more likely to have hypertension and diabetes mellitus than the control subjects. Moreover, in all studied populations, the age, body mass index (BMI), total cholesterol, and low-density lipoprotein cholesterol levels were much higher in CAD patients than in controls, and the high-density lipoprotein cholesterol level was significantly lower in CAD patients than in controls (Table 1).

**Table 1:** Clinical characteristics of the studied Chinese Han population.

Characteristics	Stage 1-discovery		*P*	Stage 2-validation		*P*	Stage 3-replication		*P*	Combined		*P*
	CAD	Control		CAD	Control		CAD	Control		CAD	Control	
Subject numbers	1,345	1,156	–	1,347	1,156	–	936	1,464	–	3,628	3,776	–
Age (years)	67.8 ± 11.4	59.2 ± 9.94	0.00	63.1 ± 11.8	61.0 ± 10.6	0.00	60.7 ± 10.4	58.1 ± 11.6	0.00	64.2 ± 11.7	59.3 ± 10.9	0.00
Gender (male, %)	58.8	61.9	0.11	73.6	53.9	0.00	54.2	66.7	0.00	63.1	61.3	0.12
Smoking (%)	23.0	22.1	0.59	31.3	14.1	0.00	36.1	25.4	0.00	29.4	20.9	0.00
BMI (kg/m^2^)	24.4 ± 0.35	24.0 ± 1.92	0.00	24.4 ± 1.28	23.9 ± 1.25	0.00	25.0 ± 2.17	24.3 ± 0.68	0.00	24.6 ± 1.39	24.1 ± 1.35	0.00
Hypertension (%)	75.2	54.4	0.00	68.0	22.5	0.00	61.4	42.1	0.00	69.0	39.9	0.00
DM (%)	29.4	24.0	0.00	22.6	8.13	0.00	15.0	7.58	0.00	23.2	12.8	0.00
Tch (mmol/L)	4.39 ± 1.01	4.28 ± 0.74	0.00	4.51 ± 1.05	4.23 ± 0.76	0.00	4.60 ± 1.19	4.39 ± 0.24	0.00	4.49 ± 1.07	4.31 ± 0.61	0.00
TG (mmol/L)	1.56 ± 1.07	1.50 ± 0.65	0.14	1.78 ± 1.04	1.48 ± 0.67	0.00	1.82 ± 1.30	1.51 ± 0.50	0.00	1.71 ± 1.13	1.50 ± 0.60	0.00
HDL-c (mmol/L)	1.11 ± 0.27	1.18 ± 0.22	0.00	1.12 ± 0.26	1.18 ± 0.20	0.00	1.19 ± 0.36	1.26 ± 0.07	0.00	1.13 ± 0.29	1.21 ± 0.18	0.00
LDL-c (mmol/L)	2.62 ± 0.83	2.46 ± 0.64	0.00	2.64 ± 0.80	2.42 ± 0.66	0.00	2.59 ± 0.67	2.41 ± 0.25	0.00	2.62 ± 0.78	2.43 ± 0.53	0.00

*The data are presented as mean ± SD or percentages*.

*CAD, coronary artery disease; BMI, body mass index; DM, diabetes mellitus; Tch, total cholesterol; TG, triglyceride; HDL-c, high-density lipoprotein cholesterol; LDL-c, low-density lipoprotein cholesterol*.

The statistic power for all the variants selected in this study was more than 80% with an effect size of 1.2 (HapMap CHB + JPT data). The statistical power was also more than 70% for the two interaction analyses (rs3806933^T^ in *TSLP* and rs6897932^T^ in *IL7R*, rs3806933^T^ in *TSLP* and rs7025417^T^ in *IL33* under the genotypic model) (23).

### Association Analysis Between Variants in TSLP, IL7R, and TSLPR and CAD

All selected variants passed the HWE test (*P* > 0.001). In stage 1, the allelic frequencies of rs3806933^T^ in *TSLP*, rs6897932^T^ in *IL7R*, and g.19646A>G^A^ in *TSLPR* were significantly different between cases and controls [rs3806933^T^ in *TSLP, P*_adj_ = 3.01 × 10^−2^, odds ratio (OR) = 1.17, 95% confidence interval (CI): 1.02–1.34; rs6897932^T^ in *IL7R, P*_adj_ = 9.64 × 10^−3^, OR = 1.26, 95%CI: 1.06–1.51; g.19646A>G^A^ in *TSLPR, P*_adj_ = 2.78 × 10^−3^, OR = 1.22, 95%CI: 1.07–1.39] after adjustment for the conventional risk factors (Table 2), however, those results did not pass the Bonferroni correction. In stage 2, the rs3806933^T^ allele of *TSLP*, rs6897932^T^ allele of *IL7R*, and g.19646A>G^A^ allele of *TSLPR* also conferred significant risk for CAD (rs3806933^T^ in *TSLP, P*_adj_ = 7.51 × 10^−4^, OR = 1.30, 95%CI: 1.11–1.50; rs6897932^T^ in *IL7R, P*_adj_ = 5.13 × 10^−4^, OR = 1.40, 95%CI: 1.16–1.69; g.19646A>G^A^ in *TSLPR, P*_adj_ = 1.52 × 10^−2^, OR = 1.19, 95%CI: 1.03–1.36) in the validation cohort, which passed Bonferroni correction. In stage 3, the associations between the three variants and CAD were confirmed in the replication cohorts (rs3806933^T^ in *TSLP, P*_adj_ = 8.89 × 10^−3^, OR = 1.20, 95%CI: 1.05–1.37; rs6897932^T^ in *IL7R, P*_adj_ = 1.71 × 10^−3^, OR = 1.33, 95%CI: 1.11–1.60; g.19646A>G^A^ in *TSLPR, P*_adj_ = 6.17 × 10^−3^, OR = 1.20, 95%CI: 1.05–1.37; Table 2), which also passed Bonferroni correction.

**Table 2:** Allelic association analysis of rs3806933 in *TSLP*, rs6897932 in *IL7R*, and g.19646A>G in *TSLPR* with CAD in the studied Chinese Han population.

Gene, SNP (Allele)	Population	*N*		Frequency		*P*_hwe_	*P*_obs_	*P*_adj_	OR (95%CI)
		Cases	Controls	Cases	Controls				
*TSLP*, rs3806933^T^	Discovery	1,207	1,104	0.354	0.317	0.673	8.27 × 10^−3^	3.01 × 10^−2^	1.17 (1.02–1.34)
	Validation	1,213	1,104	0.353	0.316	0.710	7.50 × 10^−3^	7.51 × 10^−4^	1.30 (1.11–1.50)
	Replication	919	1,361	0.392	0.344	0.332	9.78 × 10^−4^	8.89 × 10^−3^	1.20 (1.05–1.37)
	Combined	3,339	3,569	0.364	0.327	0.273	4.69 × 10^−6^	4.35 × 10^−5^	1.18 (1.09–1.27)
*IL7R*, rs6897932^T^	Discovery	1,219	1,149	0.188	0.149	0.577	3.47 × 10^−4^	9.64 × 10^−3^	1.26 (1.06–1.51)
	Validation	1,223	1,150	0.188	0.150	0.598	3.58 × 10^−4^	5.13 × 10^−4^	1.40 (1.16–1.69)
	Replication	888	1,462	0.169	0.131	0.682	3.25 × 10^−4^	1.71 × 10^−3^	1.33 (1.11–1.60)
	Combined	3,330	3,761	0.183	0.142	0.367	3.80 × 10^−11^	1.13 × 10^−7^	1.31 (1.19–1.45)
*TSLPR*, g.19646A>G^A^	Discovery	1,345	1,156	0.405	0.361	0.169	1.41 × 10^−3^	2.78 × 10^−3^	1.22 (1.07–1.39)
	Validation	1,347	1,156	0.405	0.362	0.167	2.06 × 10^−3^	1.52 × 10^−2^	1.19 (1.03–1.36)
	Replication	936	1,464	0.413	0.368	0.336	1.89 × 10^−3^	6.17 × 10^−3^	1.20 (1.05–1.37)
	Combined	3,628	3,776	0.407	0.364	0.359	8.57 × 10^−8^	2.04 × 10^−6^	1.20 (1.11–1.29)

*P_hwe_, P value from Hardy–Weinberg equilibrium tests; P_obs_, observed P value; P_adj_, P value adjusted by covariates; OR, odds ratio after adjustment; TSLP, thymic stromal lymphopoietin; IL7R, interleukin 7 receptor; TSLPR, TSLP receptor; CAD, coronary artery disease; CI, confidence interval*.

*In the combined cohorts: 3,339 CAD cases and 3,569 controls were genotyped successfully for rs3806933; 3,330 CAD cases and 3,761 controls were genotyped successfully for rs6897932; 3,628 CAD cases and 3,776 controls were genotyped successfully for g.19646A>G*.

Combining the three cohorts together, a meta-analysis with 3,628 CAD cases and 3,776 controls was performed. In the combined cohorts: 3,339 CAD cases and 3,569 controls were genotyped successfully for rs3806933; 3,330 CAD cases and 3,761 controls were genotyped successfully for rs6897932; 3,628 CAD cases and 3,776 controls were genotyped successfully for g.19646A>G. The above three variants all were significantly associated with CAD in the combined cohort (rs3806933^T^ in *TSLP, P*_adj_ = 4.35 × 10^−5^, OR = 1.18, 95%CI: 1.09–1.27; rs6897932^T^ in *IL7R, P*_adj_ = 1.13 × 10^−7^, OR = 1.31, 95%CI: 1.19–1.45; g.19646A>G^A^ in *TSLPR, P*_adj_ = 2.04 × 10^−6^, OR = 1.20, 95%CI: 1.11–1.29) after adjustment for covariates of CAD, which passed Bonferroni correction (Table 2). Significant genotypic association results for the three variants above (rs3806933^T^ in *TSLP*, rs6897932^T^ in *IL7R*, g.19646A>G^A^ in *TSLPR*) were also identified for CAD in the combined cohort under an additive model after applying Bonferroni correction (Table S2 in Supplementary Material).

The allelic association results between rs2289276 in *TSLP*, rs7737000, rs1494555, and rs1494558 in *IL7R*, and rs150166261, rs36133495, and rs36177645 in *TSLPR* and CAD were not significant with adjusted *P* values of more than 0.05 in the first stage (Table S3 in Supplementary Material). The genotypic association results also were not significant with adjusted *P* values of more than 0.05 under any one of the three models (additive, dominant, and recessive) for these variants (Table S4 in Supplementary Material). Therefore, we discarded these variants in the next two stages.

### Reporter Gene Analysis

**Figure 2:**
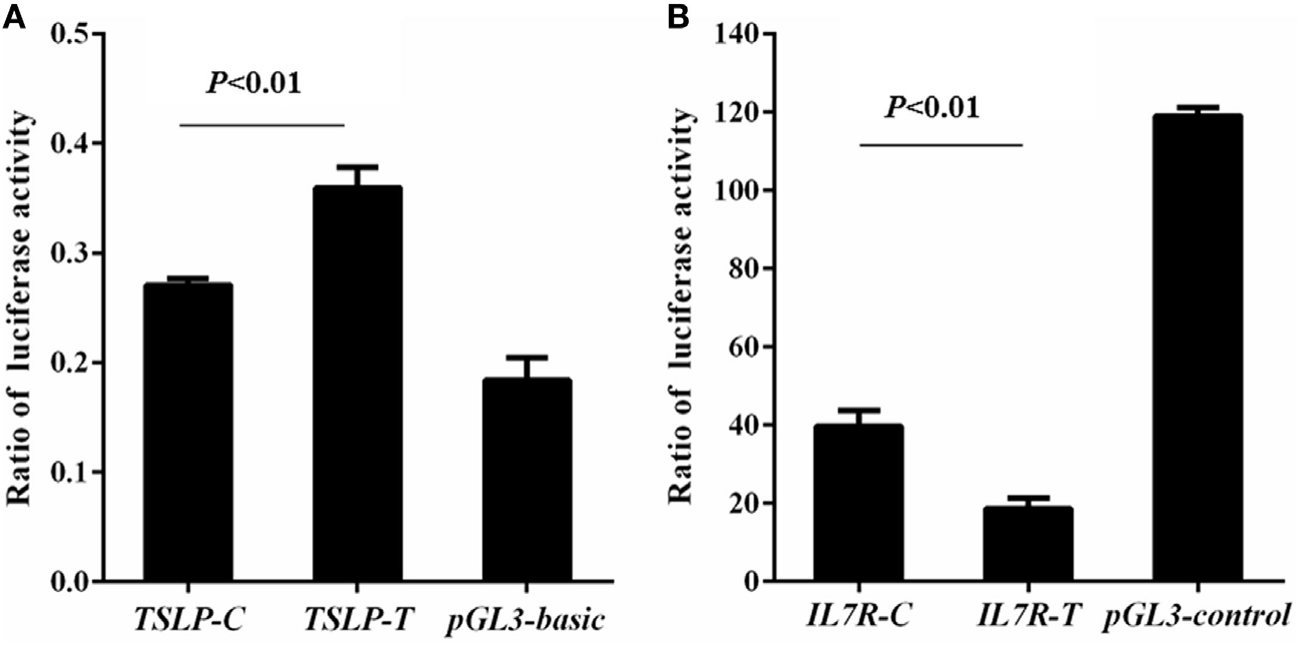
Reporter gene analysis for thymic stromal lymphopoietin (*TSLP*) **(A)** and interleukin 7 receptor (*IL7R*) **(B)**. Luciferase activity was tested by means of cellular extracts. Mean ± SD of the relative luciferase activity is shown. The difference in the luciferase activity among the reference plasmids was analyzed using the ANOVA test. The terminology of *P* < 0.01 indicates statistical significance.

Rs3806933 was located in the promoter region of *TSLP* which might regulate the expression of *TSLP*. Using luciferase assay, we found that compared to the empty pGL3-basic vector group, the luciferase activity increased in both the construct TSLP-T and construct TSLP-C groups, and the luciferase activity increased more for the construct TSLP-T than for TSLP-C (*P* < 0.01; Figure 2A). These results indicated that rs3806933^T^ might increase the expression of *TSLP*. Rs6897932, located within the alternatively spliced exon 6 of *IL7R*, had a functional effect on gene expression. Luciferase activity in the construct IL7R-T and construct IL7R-C group were decreased than that in the empty pGL3-control vector group, and plasmid with the risk allele of rs6897932 in *IL7R* (IL7R-T) showed considerably lower luciferase activity than the plasmid IL7R-C (*P* < 0.01; Figure 2B), which suggested that rs6897932^T^ might decrease the expression of *IL7R*. These results were validated in another two independent experiments. In addition, we did not find significantly different luciferase activity between the two plasmids for g.19646A>G in *TSLPR* (TSLPR-G and TSLPR-A; *P* ≥ 0.05; data not shown). It was concluded that the allele changes of rs3806933 in *TSLP* and rs6897932 in *IL7R* could both regulate the expression of their genes, whereas g.19646A>G in *TSLPR* might not.

### Association of rs3806933 Genotypes With Plasma TSLP Concentration

**Figure 3:**
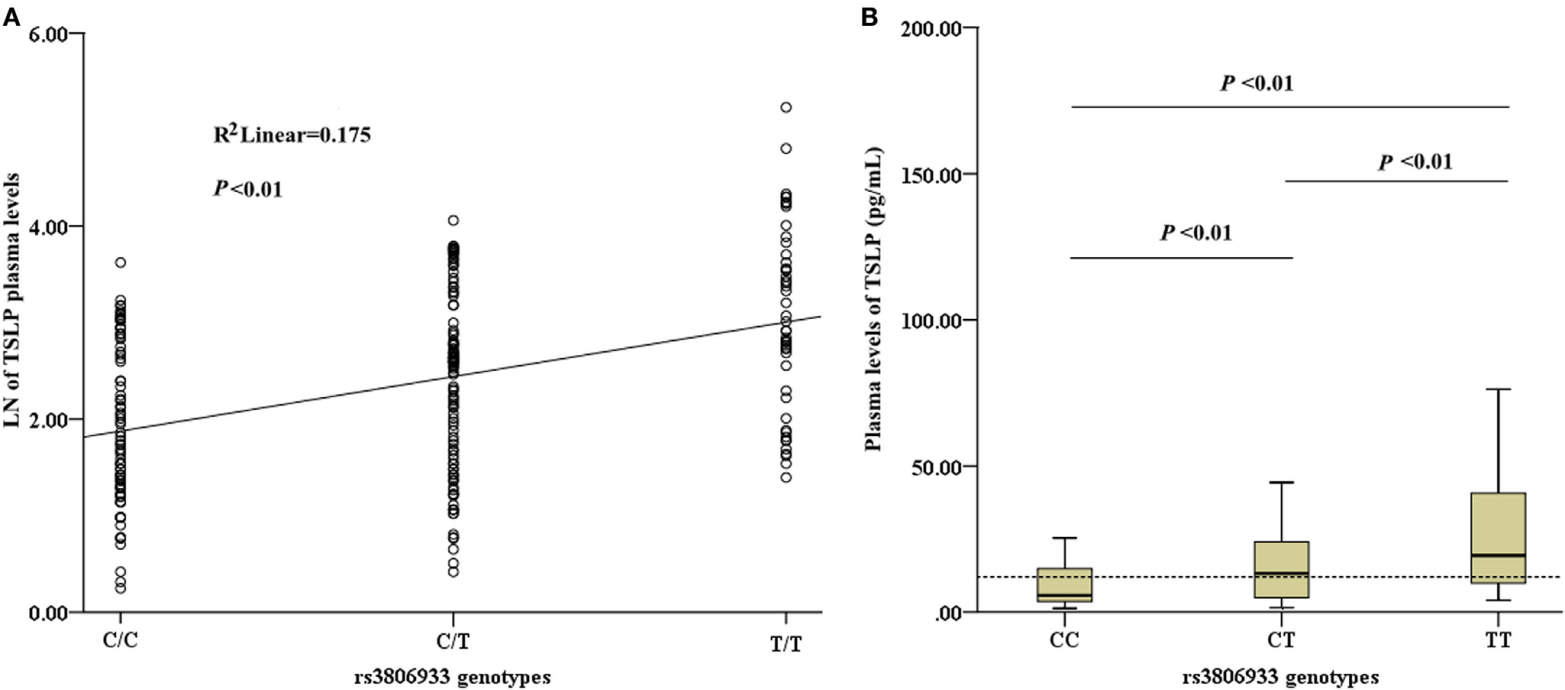
Analysis of circulating thymic stromal lymphopoietin (TSLP) level. **(A)** Association analysis between the circulating level of TSLP and the rs3806933 genotypes in a linear regression model; **(B)** Comparisons among every pair of the three genotypes, *via* the ANOVA test. The terminology of *P* < 0.01 indicates statistical significance. A broken line indicates the median value of 12.03 pg/mL of TSLP levels that were detectable in coronary artery disease patients. The extremes in the Box Graph analysis of panel **(B)** have been removed to avoid the interference.

Among the 336 CAD patients, the detection rate of plasma TSLP concentrations was 70.5% (median, 12.03 pg/mL; range, 1.28–187.04 pg/mL). All the detectable samples were included in this study. Linear regression analysis revealed a significant association between the rs3806933 genotypes and the plasma TSLP levels in the subjects with a detectable TSLP level (*n* = 237, *R*^2^ = 0.175, *P* < 0.01; Figure 3A). This trend was confirmed by the ANOVA test for the contrast among each pair of the three genotypes (*P* < 0.01; Figure 3B). These results indicated that the circulating level of TSLP could be regulated by the variant rs3806933 in *TSLP* and as the number of risk allele “T” of rs3806933 increased, the circulating level of TSLP increased.

### Interaction Association Analysis Between rs3806933 in *TSLP* and rs6897932 in *IL7R* in the Combined Chinese Han Population

Because rs3806933 in *TSLP* and rs6897932 in *IL7R* have been suggested to be functional variants by previous studies (31–33), we performed an interaction analysis between these two variants under the genotypic model in the combined Chinese Han population. In the combined cohort, 2,879 CAD cases and 3,249 controls were genotyped successfully for both rs3806933 and rs6897932. The interaction analysis showed a considerably lower *P* value (5.97 × 10^−10^) in association with CAD than the single variants did (Table 3). The combination genotype of “rs3806933_TT/rs6897932_TT” with the largest effect size provided a nearly fivefold increase in the risk for CAD (*P*_adj_ = 6.99 × 10^−4^, OR = 4.85, 95%CI: 1.95–12.2; Table 3).

**Table 3:** Interaction analysis between rs3806933 in thymic stromal lymphopoietin and rs6897932 in interleukin 7 receptor under the genotypic model in the combined population.

Population (*n*, case/control)	Types	Case (%)	Control (%)	*P*_adj_	Odds ratio (95% confidence interval)
Combined (2,879/3,249)	TT/TT	24 (0.83)	8 (0.25)	6.99 × 10^−4^	4.85 (1.95–12.2)
	TT/CT	129 (4.48)	92 (2.83)	6.06 × 10^−5^	1.88 (1.38–2.57)
	TT/CC	280 (9.73)	253 (7.79)	4.07 × 10^−4^	1.47 (1.19–1.81)
	CT/TT	48 (1.67)	30 (0.92)	6.94 × 10^−3^	2.03 (1.21–3.39)
	CT/CT	319 (11.1)	346 (10.6)	3.32 × 10^−2^	1.24 (1.02–1.50)
	CT/CC	862 (29.9)	1,029 (31.7)	2.92 × 10^−2^	0.86 (0.74–0.98)
	CC/TT	41 (1.42)	32 (0.98)	3.50 × 10^−2^	1.73 (1.04–2.87)
	CC/CT	350 (12.2)	338 (10.4)	2.68 × 10^−4^	1.43 (1.18–1.74)
	CC/CC	826 (28.7)	1,121 (34.5)	–	1
	ADD/ADD	–	–	5.97 × 10^−10^	–

*P_adj_, P value after adjustment for covariates, such as age, gender, smoking, body mass index, hypertension, diabetes mellitus, total cholesterol, triglyceride, high-density lipoprotein cholesterol, and low-density lipoprotein cholesterol; Additive model, rs3806933_CC/CT/TT, rs6897932_CC/CT/TT*.

### Interaction Association Analysis Between rs3806933 in *TSLP* and rs7025417 in *IL33* in the Combined Chinese Han Population

**Figure 4:**
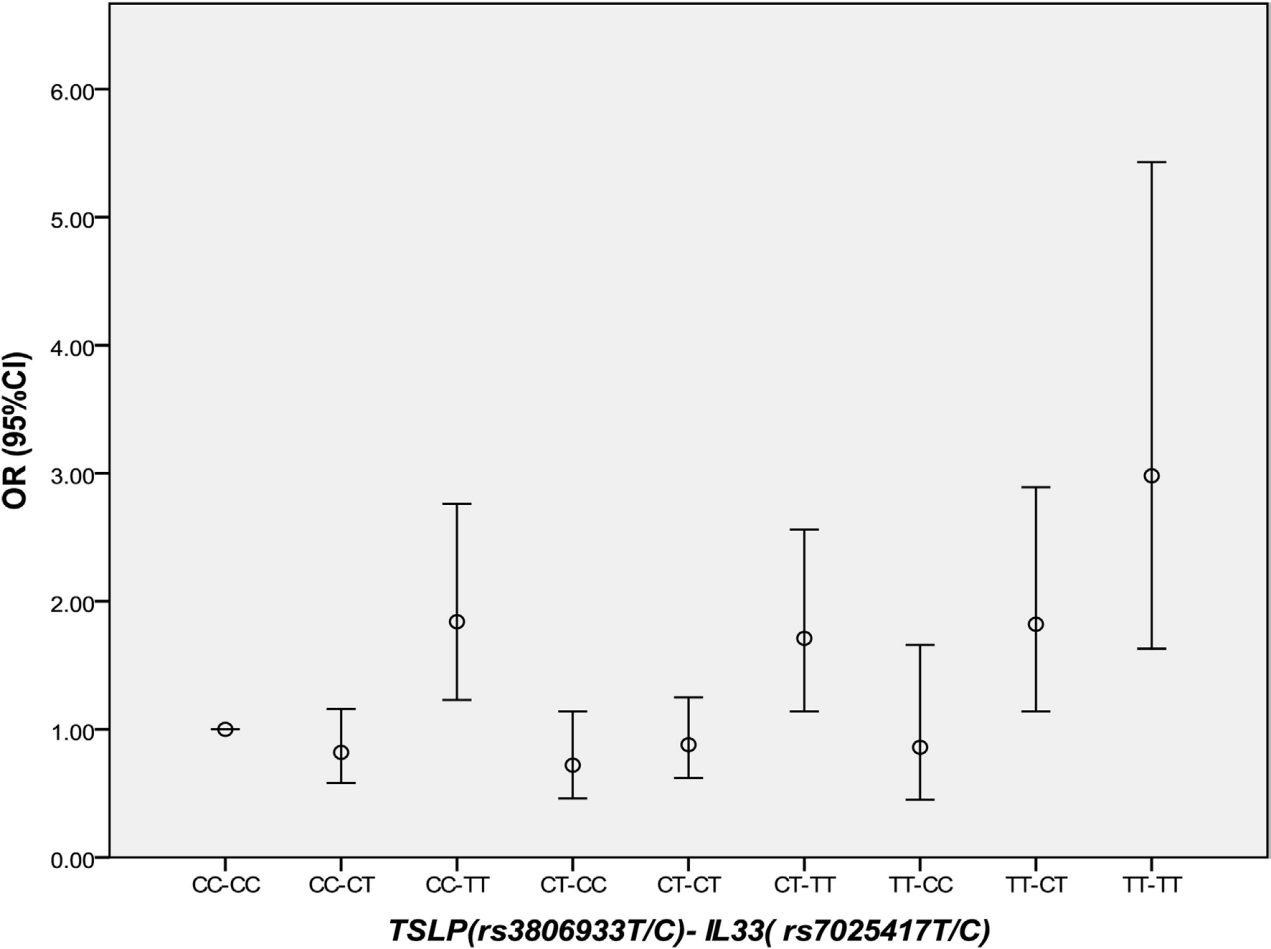
Comparison of odds ratio (OR) values for the interaction analysis between rs3806933 in thymic stromal lymphopoietin (*TSLP*) and rs7025417 in *IL33*. OR values with a 95% confidence interval (CI) for the combinations under the genotypic model.

Thymic stromal lymphopoietin and IL-33 might interact with each other in immune inflammatory diseases. However, in CAD, this has not been studied yet. It was interesting that in 2013 we found the IL-33–ST2L pathway is causally involved in the development of CAD (23). Considering that most of the same samples were used in the two studies, we further performed an interaction analysis between rs3806933 in *TSLP* and rs7025417 in *IL33* under the genotypic model in the same population. In the combined cohort, 1,736 CAD cases and 1,093 controls were genotyped successfully for both rs3806933 and rs7025417. The interaction analysis of the two SNPs (rs3806933 in *TSLP* and rs7025417 in *IL33*) indicated significant associations with a *P* value of 3.67 × 10^−4^ for the type of rs3806933_TT/rs7025417_TT, which provided a nearly threefold increase in the risk of CAD (OR = 2.98, 95%CI: 1.63–5.43; Table 4; Figure 4).

**Table 4:** Interaction analysis between rs3806933 in thymic stromal lymphopoietin (*TSLP*) and rs7025417 in *IL33* under the genotypic model in the combined population.

Population (*n*, case/control)	Types	Case (%)	Control (%)	*P*_adj_	OR (95% CI)
Combined (1,736/1,093)	TT/TT	83 (4.78)	22 (2.01)	3.67 × 10^−4^	2.98 (1.63–5.43)
	TT/CT	132 (7.60)	61 (5.58)	1.17 × 10^−2^	1.82 (1.14–2.89)
	TT/CC	40 (2.30)	27 (2.47)	6.52 × 10^−1^	0.86 (0.45–1.66)
	CT/TT	232 (13.4)	106 (9.70)	9.29 × 10^−3^	1.71 (1.14–2.56)
	CT/CT	373 (21.5)	283 (25.9)	4.74 × 10^−1^	0.88 (0.62–1.25)
	CT/CC	111 (6.39)	79 (7.23)	1.59 × 10^−1^	0.72 (0.46–1.14)
	CC/TT	222 (12.8)	104 (9.52)	3.25 × 10^−3^	1.84 (1.23–2.76)
	CC/CT	426 (24.5)	306 (28.0)	2.67 × 10^−1^	0.82 (0.58–1.17)
	CC/CC	117 (6.74)	105 (9.61)	–	1
	ADD/ADD	–	–	1.98 × 10^−3^	–

*P_adj_, P value adjusted for age, gender, body mass index, hypertension, diabetes mellitus, smoking, total cholesterol, triglyceride, high-density lipoprotein cholesterol, and low-density lipoprotein cholesterol; OR, odds ratio after the adjustment; CI, confidence interval. Additive model, rs3806933_CC/CT/TT, rs7025417_CC/CT/TT*.

### GRS Analysis

In addition, we analyzed the GRSs based on the SNPs in *TSLP* and *IL33* (GRS3, rs3806933 and rs7025417) or only an SNP in the single gene (GRS1, rs3806933 in *TSLP*; GRS2, rs7025417 in *IL33*) by a Cox regression model. Our results showed that, after adjusting for the conventional risk factors of CAD, GRS3 increased the C index by 3.1% as compared to GRS1 and by 0.3% as compared to GRS2 (Table 5).

**Table 5:** Comparison of the GRSs for prediction of CAD in the combined population.

GRS	C index (95%CI)	*P*	Comparison with GRS1 C index change	Comparison with GRS2 C index change
GRS1 (*TSLP*)	0.526 (0.50–0.55)	1.99 × 10^−2^	NA	−0.028
GRS2 (*IL33*)	0.554 (0.53–0.58)	1.06 × 10^−6^	0.028	NA
GRS3 (*TSLP-IL33*)	0.557 (0.54–0.58)	2.55 × 10^−7^	0.031	0.003

*GRS, genetic risk score; GRS1, genetic risk score based on rs3806933 in TSLP; GRS2, genetic risk score based on rs7025417 in IL33; GRS3, genetic risk score based on rs3806933 in TSLP and rs7025417 in IL33; TSLP, thymic stromal lymphopoietin; CAD, coronary artery disease; CI, confidence interval*.

## Discussion

In this study, we discovered that rs3806933 in *TSLP*, rs6897932 in *IL7R*, and g.19646A>G in *TSLPR* were significantly associated with the pathogenesis of CAD in the Chinese Han population. In addition, we confirmed that rs3806933 in *TSLP* and rs6897932 in *IL7R* could influence the expression of their genes as evidenced by a reporter gene analysis or protein circulation level study. We also found that the interaction between rs3806933 in *TSLP* and rs6897932 in *IL7R* contributed to CAD with the highest risk effect. Interestingly, the significant association result from the interaction analysis between rs3806933 in *TSLP* and rs7025417 in *IL33* indicated that the two genes of *TSLP* and *IL33* might confer an epistatic effect in the development of CAD.

In 1994, Friend et al. first discovered the murine TSLP as a new growth factor that could regulate the B and T lineage cells (34). In 2001, Reche et al. scanned the human genome database and identified its human homolog TSLP (35). Although the human and murine TSLP share only 43% amino acid homology, they have similar biological functions (9, 36). Multiple inflammatory cells, such as DCs and mast cell lines, express TSLP; TSLP can also impact a wide range of inflammatory cell lines, such as basophils, eosinophils, CD4^+^, CD8^+^, NK T cells, and B cells (37–39). Studies have reported that TSLP played important roles in inflammatory diseases, including asthma and Behçet’s disease (40, 41). The functional TSLP receptor (TSLPR subunit and IL-7Rα) is mainly expressed on monocytes and DCs, and occasionally on lymphocytes (33, 42). These studies demonstrated that the TSLP/TSLPR axis is involved in the immune system, which prompted us to investigate the role of this axis in CAD.

In our study, we discovered three genetic risk variants (rs3806933 in *TSLP* and rs6897932 in *IL7R* and g.19646A>G in *TSLPR*) for CAD. Furthermore, reporter gene analysis showed that rs3806933 in *TSLP* and rs6897932 in *IL7R* could regulate the mRNA expression of their respective genes. In addition, the circulating level study also confirmed that the variant rs3806933 in *TSLP* could influence the circulating level of TSLP protein. These results indicate that variants in the TSLP/TSLPR axis might affect the risk of CAD through upregulating mRNA or protein expression, and the variants or key genes are likely to be causal risk factors for CAD.

In 2000, Pandey et al. and Park et al. demonstrated that IL-7Rα, in combination with TSLPR, can increase the binding affinity of TSLP for its receptors, which might promote the downstream signaling of TSLP, thus improving its biological efficacy (14, 15). In 2007, Gregory et al. and Lundmark et al. both independently demonstrated that rs6897932 in *IL7R* could increase the ratio of membrane-bound IL-7Rα to soluble IL-7Rα by decreasing the amount of soluble IL-7Rα protein (32, 33). In 2009, Harada et al. demonstrated that rs3806933, which is in the promoter region of *TSLP*, can influence the binding activity of the transcription factor activating protein-1 and thereby regulate the expression of TSLP (31). In the present study, we confirmed the functional roles of rs3806933 in *TSLP* and rs6897932 in *IL7R* by reporter gene analysis and a circulating level study, which indicated that the two variants could regulate the expression of their respective genes. As the effect of the common single variant is too small to be of clinical significance, we performed the gene–gene interaction analysis and found that the OR values for the combined types of rs3806933 in *TSLP* and rs6897932 in *IL7R* were much higher than those for the individual types for the associations with CAD. Therefore, we hypothesized that the two variants (rs3806933 in *TSLP* and rs6897932 in *IL7R*) might interact with each other in a biologically relevant way and the crosstalk between TSLP and IL-7Rα might greatly enhance the risk of CAD.

A previous CAD GWAS meta-analysis showed that rs3806933 in *TSLP* is moderately associated with CAD and rs6897932 in *IL7R* is not associated with CAD in the European ancestry (43). Here, we found that rs3806933 in *TSLP* and rs6897932 in *IL7R* might be the specific cis-expression quantitative trait loci (eQTLs) for CAD in our Chinese Han population. This inconsistency between the results may be explained by the heterogeneity of the eQTL in different diseases or populations with different ancestries (44). Further functional studies for CAD in the Chinese Han population are warranted to confirm our results.

In classical Mendelian genetics, some variants may influence the effects of other variants, such as “stopping” or “standing above,” and are defined as epistatic (45). Though they exist in different pathways, these variants might interact with each other in some diseases, in which their pathways are both involved. In general, the specific composition of the alleles with an epistatic effect is determined specifically by the disease’s phenotype, and accumulating evidence (45) has demonstrated the epistatic effect of these related variants in complex diseases.

In 2013, we reported that variants in the IL-33 signaling pathway might influence the development of CAD by regulating the expression of key genes of the pathway and then influenced the risk of CAD (23). IL-33 was a strong inducer of Th2 responses and TSLP also engaged in the process of Th2 inflammation (46). In recent years, more and more studies have indicated that the TSLP and IL-33 signaling pathways might interact with each other in Th2 cell-mediated inflammatory responses (25–27). In 2015, Li et al. found that the promoter variant rs992969 in *IL33* could increase the gene protein expression and eosinophil counts in human bronchial epithelial biopsy (BEC) and then influence the risk of asthma (18). In the same year, Liao et al. studied the cross-regulation between the IL-33 and TSLP signaling pathways in human nasal epithelial cells and discovered that IL-33 could induce the expression of TSLP/TSLPR/IL-7Rα, and conversely, TSLP could induce the expression of IL-33 receptors, such as ST2L, thereby upregulating the IL-33-induced TSLP expression (47). The positive feedback loop between IL-33 and TSLP and their receptors might facilitate the Th2-skewed inflammation in eosinophilic chronic rhinosinusitis with nasal polyps (47). However, in atherosclerosis, the role of Th2 inflammation was still confused yet: when targeted deletion of Th2 cytokine, IL-5, atherosclerosis progression was accelerated, which proposed an athero-protective role of Th2 inflammation (48); when targeted deletion of Th2 cytokine, IL-4, the ApoE^−/−^ mice and LDLR^−/−^ mice developed less severe atherosclerotic, which implied a proatherogenic function of Th2 inflammation (49, 50). In this study, we not only demonstrated that variants in the TSLP/TSLPR axis regulated the expression of the key genes and influenced the risk of CAD but also discovered that the effect of the interaction between variants in *TSLP* and *IL33* was much higher than those of the single genes. Considering our findings together, we hypothesized that variants in the TSLP/TSLPR axis might regulate the expression of the key genes in the pathways as well as the cytokines and their receptors involved in the development of CAD by a positive feedback effect, which could also be called an epistatic effect. This positive feedback loop among IL-33, TSLP, and their receptors might increase CAD risk through facilitating the Th2-skewed inflammation. Further functional studies are needed to confirm our hypothesis.

In conclusion, this study indicated that the TSLP/TSLPR axis might affect the CAD risk through upregulating the reference genes’ mRNA expression or protein secretion, and the TSLP and IL-33 signaling pathways might have an epistatic effect on the risk of CAD. These results might provide a novel point of view for the prevention and treatment of CAD based on targeting the mechanisms of the epistatic effect between the related cytokines.

## Data Availability

We declare that all the data supporting the findings of this study are available within the article and the Supplementary Information files and can be obtained from the corresponding authors upon reasonable request.

## Ethics Statement

This study followed the guidelines set forth by the Declaration of Helsinki and passed the review of the Ethics Committee of Tongji Medical College, and Huazhong University of Science and Technology. All study participants signed a written informed consent form.

## Author Contributions

Conceived and designed the experiments: XT and XC. Performed the experiments and analyzed the data: S-FN, L-FZ, and QF. Contributed reagents/materials/analysis tools: S-FN, L-FZ, QF, Y-HL, H-SZ, Q-WC, FW, T-TT, NX, C-QX, J-YZ, Y-ZL, W-JZ, Z-PZ, JJ, Y-YL, TX, DW, C-CW, J-JF, H-BX, JY, QY, P-YW, S-HT, Q-LL, Q-XL, JQ, BL, GW, Y-XW, YY, X-PY, YH, QW, XC, and XT. Wrote the paper: S-FN. All authors reviewed the manuscript.

## Conflict of Interest Statement

The authors declare that the research was conducted in the absence of any commercial or financial relationships that could be construed as a potential conflict of interest.

## Abbreviations

TSLP, thymic stromal lymphopoietin; TSLPR, thymic stromal lymphopoietin receptor; IL7R, interleukin 7 receptor; CAD, coronary artery disease; GWAS, genome-wide association studies; Tregs, regulatory T cells; DCs, dendritic cells; IL-7Rα, IL-7 receptor alpha; ApoE KO, ApoE knockout mice; BMI, body mass index; ELISA, enzyme-linked immunosorbent assay; HWE, Hardy–Weinberg equilibrium; LD, linkage disequilibrium; MAF, minor allele frequency; OR, odds ratio; CI, confidence interval; GRS, genetic risk score.
