# Supplementary material for: Genetic Regulation of the Thymic Stromal Lymphopoietin (TSLP)/TSLP Receptor (TSLPR) Gene Expression and Influence of Epistatic Interactions Between IL-33 and the TSLP/TSLPR Axis on Risk of Coronary Artery Disease
Source: Front Immunol. 2018 Aug 3;9:1775. doi: 10.3389/fimmu.2018.01775 (PMC6085432; doi:10.3389/fimmu.2018.01775)
Supplement: Supplementary file 1 [file Table_1.doc]

Supplementary Material

**Genetic Regulation of the Thymic Stromal Lymphopoietin (TSLP)/TSLP Receptor Gene Expression and Influence of Epistatic Interactions between IL-33 and the TSLP/TSLP Receptor Axis on Risk of Coronary Artery Disease**

# Supplementary Data

# *Subjects enrollment*

The CAD was defined as a diameter stenosis＞70% in any of the main coronary arteries by coronary angiography, or coronary artery bypass graft, percutaneous coronary intervention, and/or myocardial infarction. Subjects with experienced myocardial spasms or had a myocardial bridge were excluded. In the discovery study, the controls were selected from subjects with the major coronary artery stenosis less than 30% confirmed by angiography and without any history of CAD. In the validation and replication studies, the controls were selected from general population under physical examinations, and without any history of potential CAD or myocardial infarction.

# *Genotyping*

Genotyping was performed in a total of 25 µL PCR volume containing 1 µL of LC Green dye, 5 pmol of each primer, 25 ng of genomic DNA, 2.5 µL of 10×PCR buffer with 1.5 mmol/L MgCl_2_, 5 mmol deoxynucleotide triphosphates, and 1 unit of Taq polymerase. Two positive controls for each genotype were included in each run. For each SNP, a total of 48 cases and controls were randomly selected for veriﬁcation of genotyping results using direct DNA sequencing analysis. DNA sequence analysis was performed with forward and/or reverse primers using the BigDye Terminator v3.1 Cycle Sequencing Kits on an ABI PRISM 3100 Genetic Analyzer (Applied Biosystems, Foster City, CA).

# *Reporter gene constructs*

A 2245bp 5'UTR sequence continuous with exon1 of *TSLP* containing the rs3806933 C allele was amplified with primer pair of 5'-CGGGGTACCCCTCCAGAAGAATGCAAGTCGAA-3'/ 5'-CCCAAGCTTACGCATAACGCGCTGTCTATCAAT-3' from a rs3806933 CC homozygous human genomic DNA sample. After separated in agarose gel, extracted and purified, the PCR products were digested with HindIII and KpnI, and then directly cloned into pGL3- basic vector. It was confirmed by restriction mapping and direct sequencing, and subsequently used as a template to generate the insert containing the T allele by using site-direct mutagenesis kit (Promega) with primers of 5'-CTAAGGTGCCCCTAGTCACCAAGAGTAGGCG-3' and 5'-CTCTTGGTGACTAGGGGCACCTTAGGGGCT-3'. It was also confirmed by restriction mapping and direct sequencing. The resulting constructs were named as TSLP-C and TSLP-T. For rs6897932, sequences containing the rs6897932^C^ allele and T allele were amplified with primer pair of 5'-cggggtaccGGAAATAATAAGTGGGCCCAC-3' and 5'-ccgctcgagCTGTGGAAATTCGCTGAGGAT-3' from a rs6897932 CC and a rs6897932 TT homozygous human genomic DNA sample respectively. For g.19646A>G, sequences containing the g.19646A>G^G^ allele and g.19646A>G^A^ allele were amplified with primer pair of 5'-CGGGGTACCGGCCGACCTGACCACACTCTCCG-3' and 5'-CCGCTCGAGCCACTGCGCCCGGCTGTTAATG-3' from a g.19646A>G GG and a g.19646A>G AA homozygous human genomic DNA sample respectively. The PCR products for rs6897932 TT/CC and g.19646A>G AA/GG were separated in agarose gel, after extracted and purified, they were digested with Xhol and KpnI, and then directly cloned into pGL3- control vector. Plasmids containing IL7R-C or IL7R-T and TSLPR-A or TSLPR-G were confirmed by restriction mapping and direct sequencing.

# *Transfection*

Cells (the human embryonic kidney cell line HEK293 and the human cervical cell line HELA) were obtained from the American Type Culture Collection (ATCC, Manassas, VA), and resuscitated and cultured according to the guidelines. HEK293 and HELA were cultured in DMEM (High Glucose) medium with 10% fetal bovine serum (FCS, GiBCO, Carlsbad, CA). When grown to 80%, they were transfected with 500 ng of each plasmid simultaneously with 50 ng of pRL-TK vector (Promega) using lipofectamine^TM^2000 (Invitrogen, Carlsbad, CA, USA). The empty pGL3-basic vector and pGL3-control vector were used as the negative control respectively.

# *Elisa*

Blood samples were collected in tubes containing EDTA as an anticoagulant. After centrifuging blood samples for 15 minutes at 1,000×g at 4°C within 30 minutes of collection, supernatant was obtained and stored at –80°C. To avoid repeated freeze-thaw cycles, detection was performed within two months. According to the manufacturer’s protocol (eBioscience Inc), enzyme-linked immunosorbent assay (ELISA) for TSLP was performed to determine the levels of TSLP protein in the plasma of the CAD. 100 uL plasma of each sample was aspirated for ELISA test and standard recombined human TSLP provided by this kit was diluted to 10 different concentrations to generate standard curves. After incubation, color development was measured at 420 nm by means of a spectrophotometer and OD value was gained for each sample. Based on the standard curve drew by the different concentrations of standard recombined human TSLP, we got the corresponding TSLP concentration of each sample. In all ELISA experiments including the following section both inter and intra assay variations were <10%, and the specificity was >70.5%.

**Supplementary Tables**

**Table S1. Allelic analyses of the *TSLPR* gene polymorphisms in CAD patients and healthy controls**

| **SNP (Allele)** | **Position** | ***P*_hwe_** | **MAF** |
| --- | --- | --- | --- |
| rs150166261^C^ | promoter | 0.040 | 0.112 |
| rs140859855^G^ | exon1 | 1 | 0.029 |
| g.6130G>A^A^ | exon3 | 1 | 0.005 |
| rs34040997^A^ | exon4 | 1 | 0.005 |
| rs191033766^A^ | intron | 1 | 0.015 |
| rs147147255T | intron | 1 | 0.034 |
| rs111884991^G^ | intron | 1 | 0.010 |
| rs9778721^G^ | intron | 1 | 0.005 |
| rs140409545^T^ | exon6 | 0.054 | 0.087 |
| rs151218732^A^ | exon6 | 1 | 0.005 |
| rs142083864^A^ | intron | 2.51×10^-5^ | 0.034 |
| rs139812396T | intron | 2.51×10^-5^ | 0.034 |
| g.19621G>A^G^ | intron | 1 | 0 |
| g.19646A>G^A^ | exon7 | 0.690 | 0.381 |
| rs36139698^T^ | exon8 | 0.420 | 0.158 |
| rs36177645^G^ | exon8 | 0.090 | 0.450 |
| rs36133495^C^ | exon8 | 0.360 | 0.470 |
| rs36158404^T^ | 3'UTR | 1 | 0.134 |

*P*_hwe_, *P* value from Hardy-Weinberg equilibrium tests; MAF, minor allele frequency.

**Table S2. Genotypic association analysis of rs3806933 in *TSLP* and rs6897932 in *IL7R* and g.19646A>G in *TSLPR* with CAD in the studied Chinese Han population**

| **Gene, SNP (allele)** | **Population**  **(n, case/control)** | **Model** | **Case (n)** | **Control (n)** | ***P*_obs_** | ***P*_adj_** | **OR (95%CI)** |
| --- | --- | --- | --- | --- | --- | --- | --- |
| *TSLP*, rs3806933^T^ | Discovery  (1207/1104) | ADD | 180/494/533 | 114/472/518 | 0.004 | 0.036 | 1.16 (1.01-1.33) |
|  |  | DOM | 674/533 | 586/518 | 0.183 | 0.335 | 1.10 (0.91-1.32) |
|  |  | REC | 180/1027 | 114/990 | 0.001 | 0.004 | 1.52 (1.15-2.02) |
|  | Validation  (1213/1104) | ADD | 180/497/536 | 113/472/519 | 0.004 | 0.002 | 1.26 (1.09-1.46) |
|  |  | DOM | 677/536 | 585/519 | 0.173 | 0.039 | 1.23 (1.01-1.51) |
|  |  | REC | 180/1033 | 113/991 | 0.001 | 0.001 | 1.69 (1.25-2.29) |
|  | Replication  (919/1361) | ADD | 152/416/351 | 169/598/594 | 0.005 | 0.010 | 1.19 (1.04-1.36) |
|  |  | DOM | 568/351 | 767/594 | 0.010 | 0.027 | 1.24 (1.02-1.49) |
|  |  | REC | 152/767 | 169/1192 | 0.006 | 0.047 | 1.30 (1.00-1.70) |
|  | Combined  (3339/3569) | ADD | 512/1407/1420 | 396/1542/1631 | 0.000 | 0.000 | 1.17 (1.08-1.26) |
|  |  | DOM | 1919/1420 | 1938/1631 | 0.008 | 0.014 | 1.14 (1.03-1.27) |
|  |  | REC | 512/2827 | 396/3173 | 0.000 | 0.000 | 1.43 (1.22-1.67) |
|  | | | | | | | |
| *IL7R*, rs6897932^T^ | Discovery  (1219/1149) | ADD | 50/359/810 | 28/287/834 | 0.002 | 0.010 | 1.26 (1.06-1.50) |
|  |  | DOM | 409/810 | 315/834 | 0.001 | 0.016 | 1.28 (1.05-1.57) |
|  |  | REC | 50/1169 | 28/1121 | 0.023 | 0.135 | 1.52 (0.88-2.61) |
|  | Validation  (1223/1150) | ADD | 51/359/813 | 28/288/834 | 0.002 | 0.001 | 1.38 (1.14-1.66) |
|  |  | DOM | 410/813 | 316/834 | 0.001 | 0.003 | 1.40 (1.12-1.74) |
|  |  | REC | 51/1172 | 28/1122 | 0.019 | 0.016 | 1.99 (1.14-3.49) |
|  | Replication  (888/1462) | ADD | 32/237/619 | 27/330/1105 | 0.001 | 0.002 | 1.33 (1.11-1.59) |
|  |  | DOM | 269/619 | 357/1105 | 0.002 | 0.008 | 1.32 (1.07-1.62) |
|  |  | REC | 32/856 | 27/1435 | 0.008 | 0.009 | 2.16 (1.21-3.86) |
|  | Combined  (3330/3761) | ADD | 133/955/2242 | 83/905/2773 | 0.000 | 0.000 | 1.30 (1.18-1.44) |
|  |  | DOM | 1088/2242 | 988/2773 | 0.000 | 0.000 | 1.32 (1.17-1.48) |
|  |  | REC | 133/3197 | 83/3678 | 0.000 | 0.000 | 1.78 (1.30-2.43) |
|  | | | | | | | |
| *TSLPR*, g.19646A>G^A^ | Discovery  (1345/1156) | ADD | 190/710/445 | 140/555/461 | 0.002 | 0.002 | 1.24 (1.08-1.42) |
|  |  | DOM | 900/445 | 695/461 | 0.000 | 0.000 | 1.42 (1.18-1.71) |
|  |  | REC | 190/1155 | 140/1016 | 0.138 | 0.410 | 1.12 (0.86-1.46) |
|  | Validation  (1347/1156) | ADD | 190/711/446 | 141/556/459 | 0.003 | 0.012 | 1.20 (1.04-1.39) |
|  |  | DOM | 901/446 | 697/459 | 0.001 | 0.010 | 1.29 (1.06-1.58) |
|  |  | REC | 190/1157 | 141/1015 | 0.160 | 0.201 | 1.20 (0.91-1.60) |
|  | Replication  (936/1464) | ADD | 159/455/322 | 207/664/593 | 0.007 | 0.006 | 1.20 (1.05-1.36) |
|  |  | DOM | 614/322 | 871/593 | 0.003 | 0.008 | 1.29 (1.07-1.55) |
|  |  | REC | 159/777 | 207/1257 | 0.058 | 0.083 | 1.24 (0.97-1.59) |
|  | Combined  (3628/3776) | ADD | 539/1876/1213 | 488/1775/1513 | 0.000 | 0.000 | 1.20 (1.12-1.30) |
|  |  | DOM | 2415/1213 | 2263/1513 | 0.000 | 0.000 | 1.34 (1.21-1.49) |
|  |  | REC | 539/3089 | 488/3288 | 0.016 | 0.066 | 1.15 (0.99-1.33) |
|  |  |  |  |  |  |  |  |
| *P*_obs_, observed *P* value; *P*_adj_, *P* value adjusted by covariates; OR, odds ratio after adjustment; ADD, additive model, rs3806933_TT/CT/CC, rs6897932_ TT/CT/CC; g.19646A>G _GG/AG/AA; DOM, dominant model, rs3806933_TT+CT/CC, rs6897932__TT+CT/CC; g.19646A>G _AG+AA /GG; REC, recessive model, rs3806933_TT/CT+CC, rs6897932_ TT/CT+CC; g.19646A>G _AA/AG+GG. In the combined cohorts: 3339 CAD cases and 3569 controls were genotyped successfully for rs3806933; 3330 CAD cases and 3761 controls were genotyped successfully for rs6897932; 3628 CAD cases and 3776 controls were genotyped successfully for g.19646A>G. | | | | | | | |

**Table S3. Allelic association analysis of other variants in TSLP/TSLP receptor axis** with CAD in the discovery population

| **Gene, SNP (Allele)** | **N** | | **Frequency** | | ***P*_hwe_** | ***P_obs_*** | ***P*_adj_** | **OR (95%CI)** |
| --- | --- | --- | --- | --- | --- | --- | --- | --- |
|  | **Cases** | **Controls** | **Cases** | **Controls** |  |  |  |  |
| *TSLP*, rs2289276^T^ | 1159 | 1032 | 0.274 | 0.271 | 0.342 | 0.821 | 0.738 | 1.09 (0.66-1.81) |
| *IL7R*, rs1494555^A^ | 1245 | 1089 | 0.449 | 0.495 | 0.215 | 0.002 | 0.719 | 0.93 (0.61-1.41) |
| *IL7R*, rs1494558^C^ | 1287 | 1024 | 0.395 | 0.435 | 0.432 | 0.006 | 0.641 | 0.90 (0.58-1.39) |
| *IL7R*, rs7737000^T^ | 1291 | 1150 | 0.160 | 0.163 | 0.708 | 0.738 | 0.543 | 0.84 (0.48-1.47) |
| *TSLPR*, rs36133495^G^ | 1060 | 970 | 0.417 | 0.404 | 0.506 | 0.370 | 0.562 | 1.04 (0.90-1.21) |
| *TSLPR*, rs36177645^G^ | 1089 | 1054 | 0.413 | 0.412 | 0.001 | 0.947 | 0.621 | 1.04 (0.90-1.20) |
| *TSLPR*, rs150166261^C^ | 1049 | 1044 | 0.112 | 0.102 | 0.182 | 0.296 | 0.280 | 1.13 (0.90-1.42) |

*P*_obs_, observed *P* value; *P*_adj_, *P* value adjusted by covariates; OR, odds ratio after adjustment; *P*_hwe_, *P* value from Hardy-Weinberg equilibrium tests.

**Table S4. Genotypic association analysis of other variants in TSLP/TSLP receptor axis** with CAD in the discovery population

| **Gene-SNP (allele)** | **N** | | **Model** | **Case (n)** | **Control (n)** | ***P*_obs_** | ***P*_adj_** | **OR (95%CI)** |
| --- | --- | --- | --- | --- | --- | --- | --- | --- |
|  | **Cases** | **Controls** |  |  |  |  |  |  |
| *TSLP*, rs2289276^T^ | 1159 | 1032 | ADD | 88/460/611 | 82/396/554 | 0.808 | 0.738 | 1.09 (0.66-1.81) |
|  |  |  | DOM | 548/611 | 478/554 | 0.652 | 0.397 | 1.32 (0.69-2.51) |
|  |  |  | REC | 88/1071 | 82/950 | 0.758 | 0.408 | 0.59 (0.17-2.07) |
| *IL7R*, rs1494555^A^ | 1245 | 1089 | ADD | 241/636/368 | 277/524/288 | 0.002 | 0.721 | 1.08 (0.71-1.63) |
|  |  |  | DOM | 877/368 | 801/288 | 0.095 | 0.182 | 1.57 (0.81-3.07) |
|  |  |  | REC | 241/1004 | 277/812 | 0.000 | 0.418 | 0.75 (0.37-1.51) |
| *IL7R*, rs1494558^C^ | 1287 | 1024 | ADD | 174/668/445 | 200/491/333 | 0.000 | 0.638 | 1.11 (0.72-1.72) |
|  |  |  | DOM | 842/445 | 691/333 | 0.299 | 0.290 | 1.41 (0.75-2.66) |
|  |  |  | REC | 174/1113 | 200/824 | 0.000 | 0.615 | 0.81 (0.35-1.85) |
| *IL7R*, rs7737000^T^ | 1291 | 1150 | ADD | 23/367/901 | 29/318/803 | 0.429 | 0.529 | 1.20 (0.68-2.22) |
|  |  |  | DOM | 390/901 | 347/803 | 0.985 | 0.507 | 1.24 (0.65-2.36) |
|  |  |  | REC | 23/1268 | 29/1121 | 0.206 | 0.909 | 1.12 (0.15-8.34) |
| *TSLPR*, rs36133495^G^ | 1060 | 970 | ADD | 198/489/373 | 163/457/350 | 0.543 | 0.571 | 1.04 (0.90-1.20) |
|  |  |  | DOM | 687/373 | 620/350 | 0.674 | 0.980 | 1.00 (0.81-1.24) |
|  |  |  | REC | 198/862 | 163/807 | 0.270 | 0.282 | 1.16 (0.89-1.51) |
| *TSLPR*, rs36177645^G^ | 1089 | 1054 | ADD | 184/531/374 | 199/470/385 | 0.143 | 0.626 | 1.04 (0.90-1.19) |
|  |  |  | DOM | 715/374 | 669/385 | 0.291 | 0.771 | 1.03 (0.84-1.27) |
|  |  |  | REC | 184/905 | 199/855 | 0.231 | 0.591 | 1.07 (0.83-1.40) |
| *TSLPR*, rs150166261^C^ | 1049 | 1044 | ADD | 9/218/822 | 7/200/837 | 0.563 | 0.270 | 1.14 (0.90-1.44) |
|  |  |  | DOM | 227/822 | 207/837 | 0.307 | 0.285 | 1.14 (0.89-1.46) |
|  |  |  | REC | 9/1040 | 7/1037 | 0.622 | 0.646 | 1.31 (0.42-4.10) |

*P*_obs_, observed *P* value; *P*_adj_, *P* value adjusted by covariates; OR, odds ratio after adjustment; ADD, additive model, DOM, dominant model, REC, recessive model.
